# Attitude and intention of migrant populations in the Netherlands regarding female genital mutilation/cutting

**DOI:** 10.1186/s12905-022-01979-5

**Published:** 2022-10-05

**Authors:** Ramin Kawous, Emily Allwood, Annemarie Middelburg, Anke van der Kwaak, Diana Geraci, Marthine Bos, Maria E. T. C. van den Muijsenbergh

**Affiliations:** 1 Erasmus University Medical Centre, Rotterdam, The Netherlands; 2 Pharos, Dutch Centre of Expertise on Health Disparities, Utrecht, The Netherlands; 3 Middelburg Human Rights Law Consultancy, Vinkeveen, The Netherlands; 4 KIT Royal Tropical Institute, Amsterdam, The Netherlands; 5 Marthine Bos Consultancy in Intercultural Competence, Deventer, The Netherlands; 6 Radboud University Medical Centre, Nijmegen, The Netherlands

**Keywords:** Attitude, Female genital mutilation/cutting, Intention, Migrants, Social pressure

## Abstract

**Background:**

Due to migration, the practice female genital mutilation/cutting (FGM/C) has become an issue of concern in high-resource countries such as the Netherlands. It was therefore of utmost importance to explore the attitude and intention of migrant populations in the Netherlands towards FGM/C, which may be leveraged to promote its elimination. Therefore, the aim of the present study was to explore the attitude and intention of migrant populations in the Netherlands regarding FGM/C.

**Methods:**

A qualitative study design was employed using Theory of Planned Behaviour (TPB) as a framework for the analysis. Data were collected using focus-group discussions (FGDs) and individual interviews. The FGDs and individual interviews were audio-recorded and transcribed *verbatim.* The main topics of the interviews were based on the constructs of TPB (attitude, subjective norms, perceived behavioral control and intention). Thus, concerning the development of categories, we opted for a hybrid form using a deductive as well as an inductive approach.

**Results:**

A total of 55 participants, 15 men and 40 women (9 born in the Netherlands) participated in the study. The findings showed that as a result of migration and regardless of country of origin and gender, many participants have changed their attitudes towards the abandonment of FGM/C. None of the participants intended to have FGM/C performed on their daughters. Generally, the social pressure to perform FGM/C seems to be lower in the Netherlands when compared to the country of origin. Most participants felt confident in their ability to resist social pressure. However, some participants feared that they might succumb to social pressure or feared that their daughters would undergo FGM/C without their consent.

**Conclusion:**

This study aimed to explore the attitude and intention of migration populations in the Netherlands regarding FGM/C. Our findings showed that the study participants had no intention to perform FGM/C on their daughters. As a consequence of acculturation process, interest in the practice of FGM/C could wane following migration. Nonetheless, some pressure to perform FGM/C still exists after migration. Newly arrived migrants and those more vulnerable to social pressure, may benefit from educational interventions that increases knowledge and awareness about various aspects of the practice, with an emphasis on empowering those individuals in facing social pressure.

## Plain English summary

Female genital mutilation/cutting (FGM/C), also known as female circumcision, is a practice which involves the cutting away of part or the whole of the external female genital organs. The practice has no health benefits, and it harms girls and women in many ways. The migration of girls and women to high-resource countries such as the Netherlands, has led to the development of programs to prevent FGM/C and to care for those affected by FGM/C.

The purpose of this study was to explore the attitude and intention of migrant populations in the Netherlands towards FGM/C. The results of this study may be relevant in the development of interventional programs to promote the elimination of FGM/C. Our findings showed that the study participants had no intention to perform FGM/C on their daughters. Many participants may have changed their attitudes towards the abandonment of FGM/C. The social pressure to perform FGM/C seems to be lower in the Netherlands when compared to the country of origin, however, some pressure to perform FGM/C still exist after migration. In this regard, educational programs are necessary, with an emphasis on empowering vulnerable groups such as newly arrived migrants in facing social pressure.

## Background

Female genital mutilation or cutting (FGM/C) refers to a cultural practice which involves ‘partial or total removal of the external female genitalia, or other injury to the female genital organs for non-medical reasons’ [1]. The World Health Organization has classified FGM/C into four major types [1] (see Table 1).

**Table 1:** World Health Organization classification of female genital mutilation/cutting [1]

Type	Definition
I	Partial or total removal of the clitoral glans and/or the prepuce/clitoral hood
II	Partial or total removal of the clitoral glans and the labia minora, with or without removal of the labia majora
III	Narrowing of the vaginal opening through the creation of a covering seal by cutting and repositioning the labia minora and/or the labia Majora, with or without removal of the clitoral prepuce/clitoral hood and glans
IV	All other harmful procedures to the female genitalia for non-medical purposes, e.g., pricking, piercing, incising, scraping and cauterizing the genital area

FGM/C is traditionally practiced in several countries in Africa, Asia, and the Middle East. As a result of international migration, the practice of FGM/C has become an issue of concern in high-resource countries, such as the Netherlands [2]. The reasons for practicing FGM/C, the extent of the practice, and type of cutting vary across ethnic groups and by geographic region. It is widely documented that justifications for supporting the practice of FGM/C are numerous and in their specific context, compelling. The practice is rooted in gender inequality and attempts to control women’s sexuality and to protect the honor of the family by ensuring virginity until marriage and martial fidelity. In addition, FGM/C would confer girls the status of eligibility for marriage [1, 3]. Globally, it is estimated that more than 200 million girls and women have undergone FGM/C [4] and nearly 68 million girls are estimated at risk between 2015 and 2030 [5].

The practice has been associated with negative health outcomes, and those with FGM/C can suffer significant, long term, and irreversible physical, psychological, and psychosexual complications [6–11]. Because of the harmful consequences to women's health and wellbeing, and the fact that FGM/C reflects deep-rooted inequality between the sexes and constitutes an extreme form of discrimination against women, the practice is internationally recognized as a human rights violation [1]. Globally, many countries have increasingly undertaken law reform to prohibit the practice [12, 13].

It is estimated that about 41,000 girls and women with FGM/C are living in the Netherlands and over 4,200 girls are at risk of being cut in the next 20 years [14]. Performing any form of FGM/C is forbidden by law in the Netherlands and punishable by a prison sentence. The Dutch approach against the practice of FGM/C focuses on preventing the practice through engagement of communities and training of (healthcare) professionals [15]. In the Netherlands, regardless of ethnicity, all children under 19 years receive general medical examinations – including genitalia up to age four—as part of a standard preventive healthcare approach performed by Youth Health Care providers. These professionals are also required to identify and assess the risk of FGM/C among migrant populations. These professionals inform parents of the health consequences of FGM/C and explain that the practice is prohibited by law. Additionally, parents are provided with a document about this law (‘statement opposing female circumcision’), signed by official authorities, to support parents in resisting pressure to have FGM/C performed when traveling abroad [16].

As a result of increasing migration, FGM/C has become a growing concern among policy makers and (health)professionals in high-resource countries such as the Netherlands [14, 17, 18]. While the attitudes of practicing populations may be leveraged to promote its elimination by developing effective policies and intervention programs, there remains a lack of knowledge regarding the attitude and intention of migrant populations in the Netherlands towards FGM/C. Moreover, professionals need to know about the practice of FGM/C in general, and understand the social dynamics that perpetuate FGM/C after migration, in order to be able to identify girls at risk and to provide culturally sensitive care to women with FGM/C.

Given the traditional nature of FGM/C, the practice has strong positive social and cultural value, which makes it difficult to eliminate the practice. Understanding factors associated with performing FGM/C could be helpful to develop effective interventions to change this behaviour. Therefore, the aim of the present study was to explore attitude and intention of migrant populations from FGM/C practicing countries in the Netherlands regarding FGM/C.

The study design was inspired by the framework of Ajzen's Theory of Planned Behavior (TPB) [19]. The TPB is one of the most prominent theories in behavior change.

The TPB proposes that behavior is predicted mostly by intentions to engage in behavior, and intentions are in turn predicted by three main variables: attitudes toward the behavior, perceived behavioral control, and subjective norms. Attitudes refer to individuals’ positive or negative evaluation of the behavior; perceived behavioral control refers to perceptions of whether the behavior is within our control; and subjective norms (social pressure) refer to perceptions of whether the ‘entourage’ (friends and family), important others, or the reference group approve of and themselves engage in the behavior.

In this study, we were particularly interested in exploring factors that may contribute to migrants’ intentions towards FGM/C. It is important to note that in this study, our aim was to explore the constructs of the TPB, rather than to examine the predictive value of these constructs in relation to migrants’ intentions to have FGM/C performed on their daughters.

Previously, most theoretical frameworks have been used to understand FGM/C at the community level (e.g., society and communities), such as Social Convention Theory [20], the Community Readiness Model [21], and the Diffusion of Innovation Model [22]. However, similar to Mackie and LeJeune [20] and Barrett and colleagues [23], we are of the opinion that beliefs and norms are better understood not only at the community levels but also at the individual level. The TPB combines elements from theories of individual behavioral change and of theories that regard FGM/C as a social convention [24].

## Methods

### Study design

A qualitative study using seven semi-structured focus group discussions (FGDs) with migrant populations from FGM/C practicing countries was carried out between June and October 2018 in the Netherlands. The Interpretative phenomenological analysis (IPA) was used to capture the experiences of the participants. The IPA focus explicitly on the links between how people describe their experiences, their cognition and their behavior. The IPA was deemed the most appropriate theoretical approach to data collection and analysis in order to understand participants’ experiences and perceptions regarding FGM/C.

Focus groups were considered an appropriate method of data collection for in-depth exploration of perspectives of participants on FGM/C, as it encouraged them to share their perspectives openly and stimulated interactions between them. Focus groups have been previously successfully used to collect data on FGM/C [25–27] and other sensitive (health) issues (e.g., [28]). In addition to the FGDs, five individual interviews were conducted to allow in-depth exploration and to ensure thematic saturation. These interviews confirmed that data saturation had been reached. This paper reports the results of this study in line with the Consolidated Criteria for Reporting Qualitative Research (COREQ) criteria. The COREQ is a checklist developed for promoting explicit and comprehensive reporting of qualitative studies and to ‘indirectly’ improve rigor, comprehensiveness and credibility of such research [29].

The study protocol was approved by the Medical Ethical Committee of Erasmus Medical Centre, Rotterdam, the Netherlands (MEC-2018-2216).

### Sampling and recruitment

Using purposive sampling, fifty-five male and female participants both first and second generation from countries where FGM/C practiced were recruited through trusted intermediaries. Of these, two participants who participated in one of our previous studies on migration and health were reapproached for participation. There was no relationship between the research team and the participants prior to commencing the study.

The recruiters were instructed to recruit participants with as even a distribution as possible among age range and gender and those who have not participated in a study on FGM/C. Unfortunately, due to limited time and resource, less male participates were recruited than female participants. The Netherlands has a significant number of migrants from very high FGM/C prevalence countries such as Somalia and Eritrea. Therefore, more people were recruited from one of these countries. The recruitment was stopped when data saturation was reached.

### Study participants

The 55 participants in this study included 15 men and 40 women from countries where FGM/C is practiced, including 9 young women born in the Netherlands to parents from an FGM/C-practicing country (so called ‘second generation’, in comparison to the ‘first generation’ participants born in their country of origin’). Ages of the participants ranged from 18 to 70, with a median age of 42 years (see Table 2). The discussions lasted between 74–120 min.

**Table 2:** Characteristics of the participants

	Focus group discussions				Individual interviews		Total
	Women 1st and 2nd generation	Men	Women, 1st generation	Women, 2nd generation	Women, 1st generation	Men	
Number of FGDs Date and duration (in minutes) of session	[1] Jun., 21 99 min	[2] Jul., 14 74–93 min	[3] Jul., 7 and 21; Sep., 21 109 -119 min	[1] Oct. 20 117 min	Sep., 8 66–75 min	Sept., 8 68 min	[7] June–October 2018
Number of participants	3	14	25	8	4	1	55
Age range	23–50	32–70	19–56	18–29	45–50	54	19–70
Number of women mentioning being cut	2	N/A	7	0	2	N/A	11
Do you have children?							
Born in the country of origin	3	15	76	0	16	0	110
Born in the Netherlands	6	13	17	0	10	0	46
Country of origin (Number of participants)	Somalia (2), Guinea (1)	Somalia (3), Ethiopia (2), Eritrea (2), Sudan (3), Egypt (1), Togo (2), Ghana (1)	Somalia (6), Egypt (1), Sudan (2), Sierra Leone (1), Eritrea (15)	Somalia (5), Eritrea (2), Egypt (1)	Somalia (1), Eritrea (1), Iraq (1), Egypt (1)	Eritrea (1)	Somalia (17), Guinea (1), Ethiopia (2), Eritrea (21), Sudan (5), Egypt (4), Togo (2), Ghana (1), Sierra Leone (1), Iraq (1)

In order for the participants to feel comfortable, we deliberately did not ask about their FGM/C-status; however, during the FGDs and interviews some women mentioned being cut. The study participants seemed to feel at ease talking about their experiences.

The average number of children in each family was 3, with one woman having 8 children. The median age of their children was 15 years. The average length of stay in the Netherlands was 12 years. The majority spoke Dutch well and were either in education or employed. Most participants frequently kept in touch with their families in their country of origin, mostly by phone or social media, and they had visited their country of origin at least once since arrival to the Netherlands. Some participants were accompanied by their children during their visit to their country of origin.

### Data collection and setting

The interview guide was inspired by the framework of the Theory of Planned Behavior (TPB) [19]. The (interview)topics included: (1) participants’ attitudes towards FGM/C, (2) perceived social pressure from others to perform FGM/C (subjective norms), (3) participants’ confidence in their ability to prevent their daughters from undergoing FGM/C (perceived behavioral control), and (4) their intention to have their daughters undergo the practice. The (almost)similar topic list for both the FGDs and interviews, was continuously fine-tuned and tailored throughout the data collection process, according to insights gained during the FGDs and interviews.

The FGDs and individual interviews were conducted in Dutch, with the exception of one FGD- and two individual interviews with participants from Eritrea which were conducted in Tigrinya with the help of professional interpreters, as the researchers did not speak Tigrinya.

As socio-cultural factors may inhibit speaking out in the presence of persons of the other sex, the FGDs were conducted among men and women separately, using same-sex moderators. During the interviews, the term ‘female circumcision’ was often used because it is the most acceptable term within the participants’ communities.

FGDs were led by four moderators (RK, EA, AvdK, MB). The moderators were either researchers or service providers with experience in FGM/C. They were accompanied by an assistant who was also an expert in the field of FGM/C. The assistants were responsible for taking notes and audio recording. The individual interviews were conducted by RK, MB, and DG.

The interviews and FGDs were held in settings that ensured privacy, most often at conference centers near the main train stations in the Netherlands. Participants were provided with lunch and refreshments before the FGDs to make them feel at ease and to create a safe space. Researchers also offered travel reimbursement and a €35 gift card to acknowledge their contributions.

All interviews and FGDs were audio-recorded and transcribed *verbatim* in Dutch.

### Data analysis

All interviews were recorded, transcribed, anonymized and uploaded into the computer software package ATLAS.ti (http://www.atlasti.com, version 8). Data were analyzed in two stages using both (I) deductive and (II) inductive analysis methods. The TPB served [19] as the explanatory background guiding the interpretation, understanding and coding of the data material. Therefore, in the first stage of data analysis, a deductive approach was applied whereby key categories were pre-determined according to the elements of the TPB (attitude, subjective norm, perceived behavioral control and intention). In the second stage, the transcripts were read through again to search for more features and patterns. An inductive analysis was then performed to identify new themes that may be not captured in the framework used.

### Trustworthiness and rigor

To ensure the trustworthiness of the data, approximately 30% of the transcriptions coded by RK were also coded by EA. Multiple coding can be a valuable strategy to assess inter-rater reliability and enhance interpretations or coding frameworks [30]. Initial differences were discussed until agreement was reached. Participants were also asked for any additional comments and/or clarifications if something was unclear. In addition, immediately after the FGDs and interviews, the moderator and assistant moderator debriefed and compared notes.

## Results

### Attitudes

#### Participants’ attitude towards the (dis)continuation of female genital mutilation/cutting

The participants were asked about their views on FGM/C and to describe the importance of FGM/C. Regardless of country of origin and gender, most participants do not support the continuation of FGM/C. Different reasons were given by the participants for not supporting the practice. They mentioned, for example that FGM/C causes severe health problems, the practice has no benefits, FGM/C is not in the best interest of the child and that it is not a religious requirement. Particularly, young female participants born in the Netherlands, described FGM/C as child abuse, and further contended that the practice was a violation of children’s rights since it is performed without their consent. For example, a 24-year-old female FGD participant born in the Netherlands (to Egyptian parents) said:

“I feel that it is an injustice to subject girls to FGM/C.”

Study participants noted that the significance of FGM/C has been changing, both in the Netherlands and in their country of origin, from being seen as a necessity to a practice that is being questioned and abandoned. Both men and women talked about the practice in terms of how FGM/C ‘used to be’ or how FGM/C was ‘in the country of origin’.

“Upon arrival in the Netherlands, it felt obvious that I would circumcise my daughter in the future, but when I learned more about the practice and about the consequences attached to it, I knew for sure that I wouldn’t subject my daughter to FGM/C, it just didn’t feel normal…”.

A 19-year-old female FGD participant from Somalia.

In one of the FGDs, female participants from Eritrea mentioned that they had experienced campaigns against FGM/C in the country of origin focusing on the harm of FGM/C and that these campaigns encourage people to question the practice.

In general, due to the sensitive and taboo nature of the practice, FGM/C is not talked about between individuals, including partners (in marriage) and their children. However, some participants noticed a change in behavior and in the discourse on FGM/C: from barely being topic of a conversation in their country of origin, they had started discussing the subject after their migration to the Netherlands. The female participants born in the Netherlands mentioned that FGM/C can be discussed between young people, but they found it difficult to discuss it with older generations.

“I have met recent migrants [in the Netherlands], I felt that it was very difficult to talk about female circumcision with them. They had young girls, circumcised, and they considered it part of culture.” A 23-year-old female FGD-participant born in the Netherlands (to Somali parents).

In general, there was a lack of knowledge and awareness among female participants born in the Netherlands regarding FGM/C. Some of them indicated that they did not know much about FGM/C before they participated in this study—they either searched online or asked their mothers about the practice.

Concerns were shared across participants about newly arrived migrants from countries where FGM/C is practiced having positive attitudes regarding FGM/C. They added that new groups need time to change their views towards the discontinuation of the practice.

### Role of adverse health consequences in the discontinuation of FGM/C

The health risks associated with FGM/C were mentioned as an important reason for supporting the discontinuation of the practice both in the country of origin as well as after the migration. According to most participants, regardless of country of origin and gender, the consequences for a girl or woman undergoing FGM/C were understood as predominantly negative. The extent of knowledge about the health consequences of FGM/C varied among participants—some had either experienced, witnessed, or were familiar with the related health consequences.

“I became familiar with the health consequences of FGM/C after migration [to the Netherlands], and I think differently about it [in a negative sense], I didn’t know anything about it back home [about the health consequences of FGM/C]”

A 50-year-old female FGD participant from Somalia.

Participants mentioned several psychological, sexual, and physical complications related to FGM/C. The impact of FGM/C on a woman’s capacity to enjoy sexual intercourse, pregnancy, and childbirth was a substantial issue for some participants.

“…it [FGM/C] had a long-term effect on my psychological wellbeing, it was painful being forced to undergo the procedure… I had psychological problems and I went to a psychologist a couple of times. I dreamt about it [FGM/C], probably because I thought about it a lot. I find it terrible for girls to undergo circumcision...and I became aware of its complications during my second delivery. A caesarean section was performed with the delivery of my daughter… I stayed 5 days at the hospital, but she was not coming out. This happened again during the delivery of my son.”

A 50-year-old female Interview participant from Iraq.

The fact that she had been subjected to FGM/C at a younger age was quite troubling for this participant from Iraq.

### Reevaluating the role of religion

Religion was mentioned as an important reason for practicing FGM/C in the country of origin, particularly in Somalia where the religion of Islam is almost universal. Female participants from Eritrea expressed that FGM/C is nowadays mainly practiced in Muslim communities in Eritrea, whereas in the past the practice of FGM/C was also dominant among Christian communities. Most participants from Somalia expressed that it is incorrect to assert that FGM/C is *‘Sunna’* in Islam; they stated that only male circumcision is *‘Sunna’* in Islam.

“I have three boys who I had circumcised, but who was I to decide to circumcise my boys, I could have left the choice up to them until they were capable of deciding for themselves…If I think about it now, if God intended men to be cut, he would have made it like that.”

A 55-year-old female FGD participant from Eritrea.

Across the FGDs, male circumcision was often brought up when participants were asked about FGM/C. They did so to make the comparison between the two practices. This female FGD participant from Eritrea (55-year-old) who migrated in the early nineties to the Netherlands, even questioned male circumcision, and pointed out the right to bodily autonomy.

## Subjective norms

### The role of social pressure on the continuation of female genital mutilation/cutting

In the context of this study, subjective norms refer to perceived social pressure from people in the Netherlands or in the country of origin to perform FGM/C. Across the FGDs and interviews, it was underlined that the social pressure and cultural significance of FGM/C in the country of origin is different compared to the Netherlands: social pressure for subjecting girls to FGM/C seems to be overall lower in the Netherlands when compared to the country of origin. After their migration to the Netherlands, they did not risk the social acceptance of their daughters nor were they concerned about the marriageability of their daughters. Moreover, with legislation outlawing the practice in the Netherlands, they felt more supported to resist social pressure to perform it. Nonetheless, the pressure to continue the practice of FGM/C emerged in some of the FGDs. A 42-year-old male FGD participant from Sudan said:

“One of my relatives, she is actually my aunt, talked with me about this [FGM/C] [by phone]. She said that they were circumcising their youngest son next week and asked me ‘what about your daughter, [name]? ’I replied, ‘What about her?’ I said, ‘No, I will not do this to her, it is a very bad thing.”

Some participants were afraid that their daughters would undergo FGM/C without their consent, for example during a holiday in the country of origin. Several participants, mainly from Somalia, Eritrea and Sudan, expressed that at some point after migration to the Netherlands they had experienced pressure from mothers, grandmothers, aunts, or mothers-in-law to have FGM/C performed on their daughters.

### Marriageability, relationships, and female genital mutilation/cutting

In the countries where FGM/C is practiced, marriageability is mentioned as one of the important reasons for supporting the continuation of the practice. Our participants described how cultural and societal norms place pressure on their communities in country of origin to perform FGM/C, to prepare their daughters for adulthood and marriage. According to participants, the practice is often considered a traditional rite of passage to womanhood, decreasing girls’ sexual desire, and thus protecting virginity, which is seen as prerequisite for marriage in the country of origin. However, while on average uncut girls may be considered ineligible for marriage in countries where FGM/C is practiced, our several female participants mentioned that men from FGM/C practicing-countries in the Netherlands prefer uncut girls (from own origin group) as their brides, especially girls who are not infibulated.

“…in my social network, most Somali boys say that they do not want to marry a circumcised girl, because of its negative [health] consequences…when I wanted to marry my husband [fiancée in the Netherlands], he said that he was not planning to marry me if I was infibulated…it felt intense [painful], everything felt gone, love, everything…”

A 39-year-old female FGD participant from Somalia.

Some female study participants also mentioned that although people often prefer to marry those with a similar background, they noticed that people are starting to establish relationships with those from other countries than the country of origin.

## Decisions and intentions to perform female genital mutilation/cutting

The participants were asked about cases of FGM/C being performed in the Netherlands and if the people living in the Netherlands intended to have their daughters cut if there was, for example no law that criminalized the practice of FGM/C. Regardless of country of origin and gender, none of the participants had the intention to have FGM/C performed on their daughters and participants were not aware of actual FGM/C cases performed in the Netherlands. However, anecdotal examples about cases of FGM/C performed elsewhere in Europe, or girls that were taken to their country of origin to undergo the practice, were provided in some FGDs; there were rumors about “cutters” that were flown into the Netherlands. Across the FGDs, some participants mentioned that if parents really wanted to practice FGM/C, they would travel during the holidays to their country of origin due to fear of prosecution in the Netherlands and possibly perform less severe forms of cutting, believing that the less severe forms will be undetectable by medical- or legal authorities in the Netherlands. Roughly half of the participants said that they would expect that more people would practice FGM/C in the Netherlands if there was no legislation prohibiting the practice.

Interviewer: “If we take a look at legislation, if circumcision would not be forbidden by law, would we see more circumcision here in the Netherlands?

Participant (50-year-old female from Somalia): Of course

Participant (23-year-old female born in the Netherlands (to Somali parents): I think so too”

At the same time, some other participants said that they expected that women who had experienced FGM/C themselves, and who truly understood the consequences of the practice, would know that the practice is wrong and would not let their daughters undergo FGM/C, whether it was prohibited by law or not.

Across the FGDs, some participants mentioned, as an unintended side-effect of the law, that some parents leave their daughters behind in the country of origin after they have been cut, while they themselves travel back to the Netherlands. Overall, the participants believed that legislation is important as it has a deterrent effect, but the mere existence of legislation is not enough to stop the practice.

## Perceived behavioral control

Participants were asked whether they could prevent their daughters from being cut (perceived behavioral control). Several participants from Somalia and Eritrea were very determined in refusing to perform FGM/C and indicated that they would easily resist social pressure from people by explaining their decisions. Social pressure made some participants recall their own experiences, and made them think about this as unjust, which triggered a change in views about the practice, creating a sense of apprehension accompanied by a sense of obligation to protect their daughters.

“When my daughter was born, my mother called me and told me to circumcise my daughter. I thought, maybe she is right. After a while, I completely changed my mind and said ‘How could you [think such a thing]?’ I said, ‘…my daughter is born here [in the Netherlands], I don’t want to hurt her, because I have been through this myself and I still have nightmares about cutting.” A 40-year-old female FGD participant from Somalia.

Some participants expressed fear about their daughters undergoing FGM/C without their consent during a visit to their country of origin. In fact, some said that they would take measures to prevent FGM/C, for instance, their children would not accompany them when visiting their country of origin, and if they were accompanied by children, they would lie about the FGM/C status of their daughters.

“If we are on vacation in our country of origin, I will lock the doors [to protect her daughters] …If people go there, just to visit [the country of origin], and when you say that you would not cut your daughter, they will then question your way of thinking. They may lie to you and cut your daughter without your consent.”

A 39-year-old female FGD participant from Somalia.

Across the FGDs, several participants pointed at the ‘statement opposing female circumcision—document’ (signed by official authorities in the Netherlands) and said that this helped them explain to family in the country of origin that FGM/C is prohibited in the Netherlands and enabled them to resist the social pressure.

## Discussion

This study aimed to explore the attitude and intention of migrant populations in the Netherlands regarding female genital mutilation/cutting (FGM/C). We explored their perceived social pressure to perform FGM/C (subjective norms) and perceived confidence in being able to prevent FGM/C (perceived behavioral control). These factors may be closely interrelated and dependent on each other and are likely to affect individuals’ decisions to engage in the practice of FGM/C [31, 32].

Our findings showed that as a result of migration, regardless of country of origin and gender, many participants have changed their attitudes towards the abandonment of FGM/C in the Netherlands. None of the participants intended to have FGM/C performed on their daughters. The adverse health consequences of FGM/C, legislation outlawing FGM/C, that the practice is not necessarily required by religion, and that the practice is as a violation of children’s rights were among the most cited reasons by our participants to not support the practice of FGM/C. The social pressure to perform FGM/C is experienced as to be overall lower in the Netherlands when compared to their country of origin. Most participants felt confident in their ability to resist social pressure; however, at the same time some participants feared that they might succumb to social pressure or feared that their daughters would undergo FGM/C without their consent, for example during a holiday in the country of origin.

The study findings echo the results of several other studies, which demonstrated that interest in the practice of FGM/C wanes following migration and that support for the continuation of FGM/C for girls born in the host country is low [33–41]. In a study, Morison and colleagues [33] investigated experiences and attitudes towards FGM/C among migrants from Somalia in the UK. They found that older generations, migrants with few signs of assimilation, and new arrivals are more likely to hold traditional views towards FGM/C. Intentions to have their daughters undergo FGM/C varied significantly with the time spent in the UK—the more integrated people were, the less likely they were to uphold traditional views towards FGM/C.

Support for the abandonment of the practice in the diaspora can be a result of acculturation. Acculturation is a process by which cultural and psychological changes occur through the contact between two or more cultural groups and their individual members [42]. Although migrants carry their cultural heritage with them when they migrate, at some point during their residence the process of acculturation occurs, and they adapt to their host country’s culture. It is important to note that acculturation effects may need time to consolidate. There is evidence to show that the amount of time spent in the host country is associated with increasingly negative attitudes towards FGM/C, and that newly arrived migrants may have more positive attitudes towards the practice [26, 37, 38, 43]. This is not surprising since the more newly arrived migrants come from an environment where FGM/C still is a social norm, and not conforming to the norm could result in social exclusion, stigma, and the inability to find a suitable marriage partner [44].

In Netherlands, as in many other host societies, the practice is uncommon and criminalized by law. According to our participants legislation against FGM/C is critical as it decreases support for the continuation of FGM/C among migrant populations. Indeed, to date, no one suspected of FGM/C has been convicted by a court in the Netherlands [45]. The enactment and enforcement of a law criminalizing FGM/C can challenge existing social norms by providing legitimacy to new sets of behaviors [44]. There is evidence to suggest that people may abandon FGM/C in an environment with legislation outlawing the practice, even if they favor the continuation of FGM/C [33–38]. Conversely, we believe that individuals from migrant populations with a more favorable attitude towards FGM/C, those with less or no confidence in their ability to prevent their daughters to undergo FGM/C, and/or those who perceive more social pressure from others, may be more likely to perform FGM/C on their daughters, regardless of the legislation outlawing the practice. As an unintended side-effect of the law in the host country, according to our participants, parents with more traditional views towards FGM/C could even leave their daughters behind in the country of origin, suggesting the fear of not conforming to the social norm may be stronger for some individuals than the fear of prosecution in the host country. It is thus important that legislation is accompanied by other preventive measures to protect girls at risk.

Further, marriageability, indirectly linked to preserving virginity, is to date among the more common or even the primary reason in some communities to perform FGM/C [44]. However, our study participants were not concerned over marriage prospects as a justification for FGM/C. They noticed that men from FGM/C practicing countries in the Netherlands prefer uncut girls (e.g., from own origin group), and that people are starting to establish relationships with those from other countries. This finding could be regarded as a sign of acculturation and in line with previous studies suggesting that migrants from FGM/C practicing countries are often relatively positive about uncut girls [26, 37, 38].

In general, as part of acculturation process, more interactions with members of other social groups in the host country who do not share expectations regarding FGM/C, may contribute to a broader shift in perceptions of societal norms among migrant populations from FGMC practicing countries. For instance, male circumcision is common in several countries where FGM/C is concentrated (40,41); our participants often talked about male circumcision when asked about FGM/C and one of our participants even questioned the practice of male circumcision and pointed out a child's right to bodily autonomy. While this one single case is not sufficient to make a generalized conclusion, it is reasonable to assume that due to acculturation, shifting opinions among some migrants could go beyond the practice of FGM/C.

Social norms or subjective norms surrounding FGM/C and perceived behavioral control are important determinants of decisions to engage in the practice of FGM/C. While social pressure to perform FGM/C still occurs after migration, similar to previous studies [34, 40, 46, 47], we found that the pressure seems to be overall lower in the host countries such as the Netherlands when compared to the country of origin, and that most migrants report being confident in resisting social pressure. Migrant populations might differ from their counterparts in the country of origin in terms of social pressure. Their counterparts might succumb to social pressure and conform to what they believe others expect of them, however, migrant populations experience overall less social pressure in the host country, hence they may experience less fear of social exclusion for not performing FGM/C. Perceived behavioral control is strongly related to social norms or subjective norms. The more support and the lower pressure to perform FGM/C, the greater individuals’ perceived behavioral control and the stronger their intention to not engage in the practice of FGM/C. A large share of our participants were determined to refuse to have FGM/C performed on their daughters and they were not afraid to protect their daughters. The ‘statement opposing female circumcision’ was highlighted as an important source of support by several participants. This document is signed by official authorities in the Netherlands to support parents in resisting pressure to have FGM/C performed when traveling abroad. Also, participants’ knowledge and awareness about various aspects of FGM/C and especially their negative experiences with FGM/C could be regarded as helpful to resist pressure and to protect their daughters.

### Limitations

As with many field studies, this study has limitations. First, despite our efforts to establish a rich and varied sample, our recruitment strategy may have resulted in over-representation of migrants with less favorable attitudes towards FGM/C. Specifically, the fact that some participants were recruited through a trusted intermediary with a clear position against FGM/C may have created a sample bias. However, given the difficulties inherent in research on the topic of FGM/C, the current study does provide much needed information on FGM/C among little studied migrant groups in the Netherlands.

Second, due to sensitivity of the topic and the illegal status of the practice in the Netherlands, participants may have provided socially desirable answers during the FGDs and interviews. Nonetheless, although migrants may be unwilling to disclose their intention to perform FGM/C, data on attitudes regarding FGM/C may be more reliable than intention [24].

Third, although TPB is the most prominent theories in behavior change and it combines elements from theories of individual behavioral change and of theories that regard FGM/C as a social convention, actual behavior may be influenced by more factors than included in the TPB, so the application of this theoretical framework may have limited our views. Nonetheless, our aim was to explore the constructs of the TPB rather than to examine the predictive value its constructs have in relation to migrants’ intentions to have FGM/C performed on their daughters.

### Conclusions and recommendations

Our findings showed that the study participants had no intention to perform FGM/C on their daughters. As a consequence of acculturation process, interest in the practice of FGM/C could wane following migration. Nonetheless, some pressure to perform FGM/C still exists after migration. It is imperative for healthcare professionals involved in the care and/or prevention of FGM/C to develop cultural competence and cultural sensitivity in the face of an increasingly diverse migrant population. Professionals should not only address the medical needs of women with FGM/C, but also broaden their scope to include their psycho-sexual and social needs. Specifically, professionals should pay more attention to family dynamics, as social pressure to perform FGM/C could be an important stressor for migrants. As their daughters grow, some parents may succumb to social pressure; therefore, they may require support in facing this pressure while attempting to safeguard their family bonds.

More research is needed to better understand how attitudes towards FGM/C wane following migration, and factors influencing the decline in the practice. Much could be gained from educational interventions with an emphasis on empowering vulnerable groups such as newly arrived migrants in facing social pressure while attempting to safeguard their family bonds. Individuals are more likely to resist social pressure and deviate from a particular social norm if they perceive more behavioral control and when their personal agency is strong [48]. The current study was not able to determine how empowered individuals differed from those less empowered individuals to protect their daughters. Future research is needed to thoroughly explore perceived behavioral control in relation to FGM/C.

## Data Availability

Data, the anonymous transcripts used during the current study are available from the corresponding author upon a reasonable request that does not contravene the informed consent forms signed by the participants.

## Abbreviations

FGD: Focus-group discussion
FGM/C: Female genital mutilation or cutting
TPB: Theory of Planned Behavior
